# Editorial: Cancer Therapeutics: Targeting DNA Repair Pathways

**DOI:** 10.3389/fmolb.2022.858514

**Published:** 2022-02-15

**Authors:** Amila Suraweera, James A. L. Brown, Yi Chieh Lim, Martin F. Lavin

**Affiliations:** ^1^ Centre for Genomics and Personalised Health, School of Biomedical Sciences and Translational Research Institute, Queensland University of Technology (QUT), Brisbane, QLD, Australia; ^2^ Princess Alexandra Hospital, Woolloongabba, QLD, Australia; ^3^ Department of Biological Sciences, University of Limerick, Limerick, Ireland; ^4^ Danish Cancer Society Research Centre, Copenhagen, Denmark; ^5^ UQ Centre for Clinical Research (UQCCR), The University of Queensland, Brisbane, QLD, Australia

**Keywords:** DNA repair, genomic, stability, double strand break, repair, cancer, therapeutics, novel chemotherapeutics

A cell’s genome is constantly challenged by exogenous and endogenous DNA damaging agents and failure to repair this damage can lead to genomic instability and tumorigenesis (Lavin et al., 2005; Jackson and Bartek, 2009). Genomic instability is an established hallmark of cancer and as tumorigenesis progresses, genetic streamlining leads to dysregulation of DNA repair pathways, selecting for cancer cells that have enhanced genomic instability and fitness. Importantly, this tumor evolution commonly leads to dependency on a single DNA repair pathway for survival through inactivation of alternate pathways, highlighting a key molecular weakness of cancer cells (Jeggo et al., 2016). Exploiting this weakness by accurately targeting the remaining or dysregulated DNA repair pathways in cancer cells using the next generation of precision/personalized medicine drugs, provides a therapeutic approach tailored to an individual’s specific tumor profile (Aziz et al., 2012; Kelley et al., 2014; Jekimovs et al., 2014; Biau et al., 2019; Lavin and Yeo, 2020).

This Research Topic explores *Cancer Therapeutics: Targeting DNA Repair Pathways* and features ten articles that reflect the breadth and complexity of DNA repair pathways used by tumors and offers key insights into their potential exploitation for the next generation of cancer therapies. This topic consists of reviews and original research articles on key DNA repair proteins and pathways that are critical for cancer development or survival, highlighting their future importance for targeted therapies.

This topic highlights significant reviews, with the manuscript by Ren et al. discussing the structure of the regulator of chromosome condensation 1 (RCC1), a protein involved in the regulation of the cell cycle, DNA damage and in the development of cancer. RCC1 is overexpressed in cancer cells and the role of RCC1 in spindle formation, nuclear envelope formation and in nuclear transport is discussed. The authors highlight the role of RCC1 in tumorigenesis and further discuss its potential as a tumor biomarker (Ren et al.). The review *Regulator of Chromosome Condensation 2 Modulates Cell Cycle Progression, Tumorigenesis, and Therapeutic Resistance*, highlights the function of RCC2 in tumor development in different cancers and its role in resistance to current therapeutics. Guo et al. demonstrate that RCC2 functions in the oncogenesis of many cancers including colorectal, lung, breast and ovarian. The authors discuss an emerging role for RCC2 in the DNA repair process. The authors suggest that interaction of RCC2 with numerous signalling pathways leads to therapeutic resistance and poor cancer outcomes in patients, highlighting its potential as a cancer biomarker and future therapeutic target. The review by Sobanski et al. *Cell Metabolism and DNA Repair Pathways: Implications for Cancer Therapy* focuses on the dependence of DNA repair on cellular metabolism. The authors highlight the interplay of DNA repair and cell metabolism in tumor development and progression, and discuss how the next generation of potential novel therapies will target both processes concurrently (Sobanski et al.). Fernandez et al. provide a comprehensive review on epigenetic therapies currently in clinical trials or FDA approved for clinical use in cancer therapy, in their review *Epigenetic Mechanisms in DNA Double Strand Break Repair: A Clinical Review*. The authors focus on the role of histone deacetylase and DNA methyltransferase inhibitors as targeted therapies and their clinical use alone or in combination with current therapies, to highlight the emerging role of these epigenetic therapies as the next wave of personalized cancer medicine (Fernandez et al.).

This topic highlights research articles investigating new biomarkers. In the article *PAXX, Not NHEJ is an Independent Prognosticator in Colon Cancer* Arora et al., performing a comprehensive analysis of several essential genes, the authors studied their association with molecular and pathological features in colon cancer. In this study, high *PAXX* expression was shown to be the only independent prognostic biomarker for disease specific and overall survival in colon cancer, amongst the non-homologous end-joining genes analysed. The authors further suggest that PAXX may represent a novel therapeutic target in colon cancer, in addition to its use as a biomarker in colon cancer. Bian et al. set out to identify the N6-methyladenosine (m^6^A) RNA methylation regulator-based prognostic signature, using the TCGA datasets, for hepatocellular carcinoma. Using a combination of bioinformatics and experimental data, the authors showed that m^6^A regulator, YTHDF1, is an oncogenic gene in hepatocellular carcinoma and functions as both a prognostic biomarker and therapeutic target in this type of cancer (Bian et al.). In order to diagnose prostate cancer (PCa) patients with a high risk of biochemical recurrence, Long et al., used a bioinformatics approach to profile DNA repair genes. The authors identified a DNA repair gene signature, developing a prognostic nomogram for PCa, accurately predicting biochemical recurrence in PCa patients. Their research highlights the importance of such predictive models for personalized treatment options and decision making in PCa.

Liu et al. demonstrated that mini-chromosome maintenance protein 4 (MCM4) is a potential biomarker in soft-tissue sarcoma which correlates with clinical staging and survival outcome in patients. Tumors with overexpression of MCM4 were therapeutically sensitive to PARP inhibitors. This highlights the potential of PARP inhibitors (alone or in combination with chemotherapy) to treat soft-tissue sarcoma patients with MCM4 overexpression. Buck et al. investigated whether veliparib, a PARP inhibitor, could enhance the radiosensitivity of medulloblastoma cells. The author’s *in vitro* data corroborated *in vivo* data from an orthotopic implant model of medulloblastoma, demonstrating that combination therapy of veliparib and irradiation decreased tumor growth rates and increased intra-tumoral apoptosis of medulloblastoma (Buck et al.). Al-Asmari et al. described the pharmacological inhibition of the STING pathway in cancer cell lines. The authors showed that induction of DNA damage in cancer cells leads to a rapid IL-6 response via non-canonical and canonical STING signalling. Furthermore, the authors suggest that targeting ERK1/2 may lead to a better response in patients, by reducing IL-6 production, whilst maintaining the anti-tumor activity of STING interferon signalling.

Together, this collection of manuscripts highlights the growing understanding and use of DNA repair pathways as both cancer biomarkers and for new precision medicine-based targeted therapeutic approaches for cancer treatment. This is particularly exemplified here by the manuscripts exploring PARP inhibitors, which we anticipate will be of interest to researchers and clinician-scientists alike. Advances in exploiting personalised profiling of tumors, combined with new biomarkers and targeted therapeutics, have, and will continue to improve clinical outcomes in patients.
